# Cavity Closure of 2-Hydroxypropyl-β-Cyclodextrin: Replica Exchange Molecular Dynamics Simulations

**DOI:** 10.3390/polym11010145

**Published:** 2019-01-16

**Authors:** Khanittha Kerdpol, Jintawee Kicuntod, Peter Wolschann, Seiji Mori, Chompoonut Rungnim, Manaschai Kunaseth, Hisashi Okumura, Nawee Kungwan, Thanyada Rungrotmongkol

**Affiliations:** 1 Department of Chemistry, Faculty of Science, Chiang Mai University, Chiang Mai 50200, Thailand; khanittha.view@gmail.com; 2 Structural and Computational Biology Research Unit, Department of Biochemistry, Faculty of Science, Chulalongkorn University, Bangkok 10330, Thailand; jintawee.ki@gmail.com (J.K.); karl.peter.wolschann@univie.ac.at (P.W.); 3 Department of Pharmaceutical Chemistry, University of Vienna, Vienna 1090, Austria; 4 Institute of Theoretical Chemistry, University of Vienna, Vienna 1090, Austria; 5 Institute of Quantum Beam Science, Graduate School of Science and Engineering, Ibaraki University, 2-1-1 Bunkyo, Mito, Ibaraki 310-8512, Japan; seiji.mori.compchem@vc.ibaraki.ac.jp; 6 National Nanotechnology Center (NANOTEC), National Science and Technology Development Agency (NSTDA), Pathum Thani 12120, Thailand; chompoonut@nanotec.or.th (C.R.); manaschai@nanotec.or.th (M.K.); 7 Research Center for Computational Science, Institute for Molecular Science, Okazaki, Aichi 444-8585, Japan; hokumura@ims.ac.jp; 8 Center of Excellence in Materials Science and Technology, Chiang Mai University, Chiang Mai 50200, Thailand; 9 Ph.D. Program in Bioinformatics and Computational Biology, Faculty of Science, Chulalongkorn University, Bangkok 10330, Thailand; 10 Molecular Sensory Science Center, Faculty of Science, Chulalongkorn University, 254 Phayathai Road, Patumwan, Bangkok 10330, Thailand

**Keywords:** 2-hydroxypropyl-β-cyclodextrin (HPβCD), replica exchange molecular dynamics (REMD), conformational change, cavity self-closure

## Abstract

2-Hydroxypropyl-β-cyclodextrin (HPβCD) has unique properties to enhance the stability and the solubility of low water-soluble compounds by inclusion complexation. An understanding of the structural properties of HPβCD and its derivatives, based on the number of 2-hydroxypropyl (HP) substituents at the α-d-glucopyranose subunits is rather important. In this work, replica exchange molecular dynamics simulations were performed to investigate the conformational changes of single- and double-sided HP-substitution, called 6-HPβCDs and 2,6-HPβCDs, respectively. The results show that the glucose subunits in both 6-HPβCDs and 2,6-HPβCDs have a lower chance of flipping than in βCD. Also, HP groups occasionally block the hydrophobic cavity of HPβCDs, thus hindering drug inclusion. We found that HPβCDs with a high number of HP-substitutions are more likely to be blocked, while HPβCDs with double-sided HP-substitutions have an even higher probability of being blocked. Overall, 6-HPβCDs with three and four HP-substitutions are highlighted as the most suitable structures for guest encapsulation, based on our conformational analyses, such as structural distortion, the radius of gyration, circularity, and cavity self-closure of the HPβCDs.

## 1. Introduction

**Figure 1:**
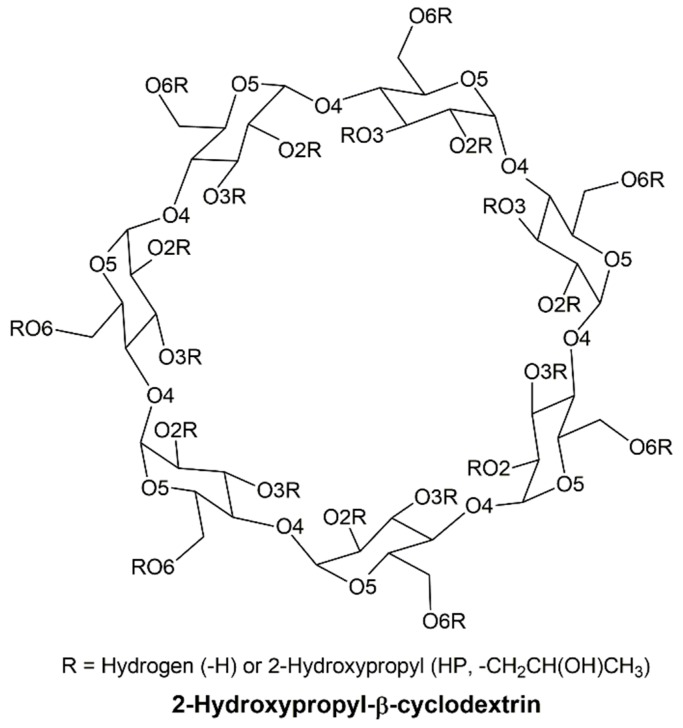
Schematic representation of 2-hydroxypropyl-β-cyclodextrin (HPβCD), which comprises seven glucopyranose units.

Cyclodextrins (CDs) are cyclic oligosaccharides, which received much attention in various technical applications, due to their unique properties. In the pharmaceutical industry, they are widely used to improve the stability and the solubility of insoluble drugs in water or organic solvent–water mixtures by molecular encapsulation [1,2,3,4,5,6,7,8,9]. The ability of the encapsulation of CDs with drugs strongly depends on the structural nature of the applied CDs. There are several different kinds of CDs that are defined by number of glucose units, and the most common CDs are α-, β-, and γ-CD, with different number of α-d-glucopyranose units (α = 6, β = 7 and γ = 8). Among these CDs, a derivative of β-cyclodextrin (βCD) named as 2-hydroxypropyl-β-cyclodextrin (HPβCD), shown in Figure 1, has been found to be more soluble and to have a lower toxicity than βCD [10,11,12,13].

HPβCD, a partially substituted poly(hydroxypropyl) ether of βCD, is commercially available as a mixture with a certain range of degrees of substitution [11,13,14,15,16]. The separation of the individual derivatives and isomers from each other is rather difficult, in particular, at the industrial scale. Also, a selective synthesis of the individual isomer is not easy; therefore, a characterization of various derivatives with regard to their inclusion ability is not possible. However, theoretical studies, particularly molecular dynamics simulations, have become a popular tool for providing this important information. By investigating the molecular structural behavior at the atomistic level of each substituted derivative, compounds with a certain degree of substitution of HPβCD can be evaluated. Note that in the technical product, the degree of substitution exists randomly, with different numbers of HP at other positions (e.g., at different glucose units at the 2, 3, and 6 positions), which depends on the concentration of the alkaline solution during the synthesis. Normally, βCD alkylations are observed at the O2 and O6 positions [13,17,18]. Therefore, HPβCD with substitutions at O2 (the most acidic position) and O6 (the most accessible position) with different degrees of substitution have been intensively investigated [11,13,14,15,16,19,20,21,22].

Generally, it is difficult to explore the conformations of biomolecules with complicated free energy surfaces and a larger number of local minima by single conventional simulation at a low temperature condition. To overcome this problem, the replica-exchange molecular dynamics simulations (REMD) [23,24], which is one of the most effective methods with generalized-ensemble algorithms, is applied by non-interacting replica exchange at various temperatures [23,24,25,26,27]. This could allow us to obtain the information of the temperature dependence. In the present study, we applied the REMD method on βCD and HPβCDs models to study the conformational change affected by the different numbers of HP-substitutions on the O2 and/or O6 atoms. The structural behaviors of all models were analyzed based on structural distortion analysis, the radius of gyration, circularity, and the cavity self-closure of the HPβCDs. By doing so, the best candidate of the HPβCDs with a suitable amount of HP-substitution, representing the perfect conical shape for molecular encapsulation, will be provided as a useful guideline for the suitable substitution degree of HPβCD.

## 2. Computational Methods

**Figure 2:**
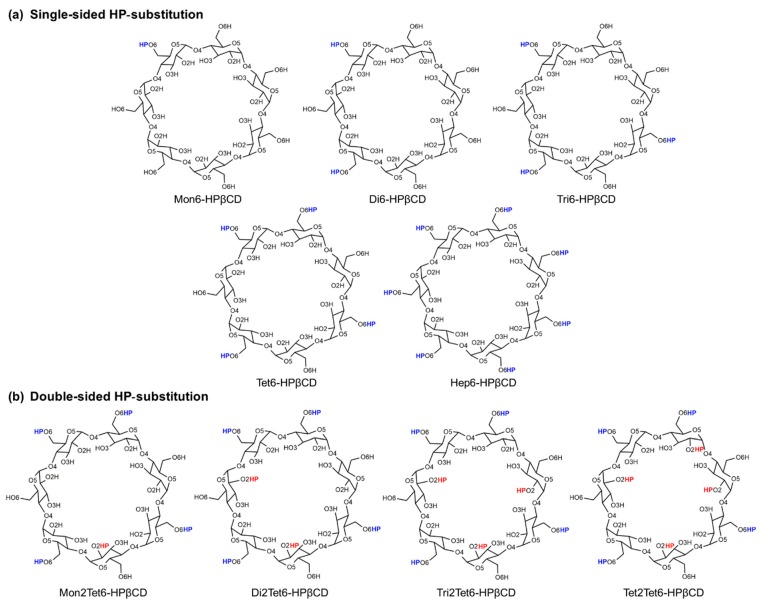
Models introducing 2-hydroxypropyl (HP) groups at the O2 (red) and/or O6 (blue) positions on glucose subunits.

The optimized structures of native βCD and Hep6-HPβCD were taken from our previous studies [28,29]. The other HPβCD derivatives were prepared by different numbers of 2-hydroxypropyl (HP) substitutions at the O2 or O6 positions on α-d-glucopyranose units with substitution degrees of around 0.14–1.14 from one to eight HP-substitutions on βCD, as shown in Figure 2. The HP-substitutions on βCD in this work were divided into two groups, single- and double-sided substitutions. For the single-sided HP-substitution (Figure 2a), the HP groups were substituted with from one up to seven HP, only on O6 atoms of the primary rim (called as 6-HPβCDs), because the O6 is more reactive compared to O2 atoms of the secondary rim. In the case of double-sided HP-substitution (Figure 2b), the structures were generated by introducing from one up to four HP groups at the O2 atoms of the non-substituted glucose units (called as 2,6-HPβCD), as defined in Table 1.

Overall, 10 CD structures were generated, to study how different numbers of HP influence the structural behavior, using REMD simulations. A detailed information of the REMD method is given elsewhere [23,24]. The REMD simulations were performed by the Amber 14 package [30]. The parameters of βCD and HPβCDs were taken from the Glycam06 carbohydrate force field [31,32], with the solvation model based on the generalized Born (GB) implicit solvent model, Igb5, which gives a suitable description of cyclodextrin εCD, as reported by Khuntawee et al. [33], and the smaller sizes of CDs (αCD-δCD, unpublished data) relative to the available crystal structures and MD studies in the explicit solvent model. The initial structures of βCD and all HPβCDs were fully minimized with 2000 steps of the steepest descent method, followed by 1000 steps of the conjugated gradient method to relax the structures before simulation. The REMD simulations were performed for 30 ns per replica, including an equilibration step for 5 ns, and the conformations at all temperatures were collected every 1 ps for 25 ns. The temperature distribution and the number of replicas were tested to obtain a reasonable replica exchange simulation. The overlapping between the potential energy distributions of eight replicas is shown in Figure S1 of the Supplementary Materials. The results confirm that the temperature from 269.5 K to 570.9 K, with interval steps of around 30–60 K, was proper for the present case.

**Figure 3:**
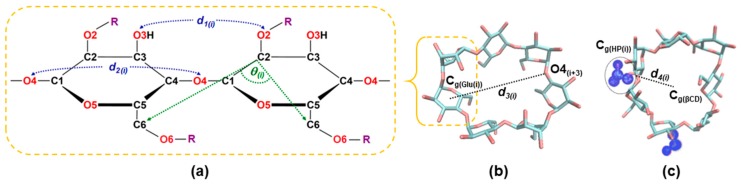
(**a**) Cyclodextrin (CD) fragment showing the atomic labels and important structural parameters, *d_1(i)_*[O2_(i)_–O3_(i+1)_], *d_2(i)_*[O4_(i)_–O4_(i+1)_], and *θ_(i)_*[C6_(i)_–C2_(i+1)_–C6_(i+1)_]. (**b**) Set of diameters, *d_3(i)_*[C_g(Glu(i))_–O4_(i+3)_], for circularity. (**c**) Set of *d_4(i)_*[C_g(βCD)_–C_g(HP(i))_] for studying cavity self-closure.

The structural distortions of all HPβCDs were analyzed by distances analysis, as defined in Figure 3. Firstly, the distances of adjacent glucopyranose units, *d_1(i)_*[O2_(i)_–O3_(i+1)_] was defined as the distance between the secondary hydroxyl groups related to the intramolecular hydrogen bonds of the wider CD rim, as labeled in Figure 3a. Secondly, the distance between the glycosidic oxygen atoms, *d_2(i)_*[O4_(i)_–O4_(i+1)_] is calculated for monitoring the ellipticity of the CDs. The probability distributions of the *d_1(i)_* and *d_2(i)_* were calculated using Equation (1) in terms of free energy:

(1) $$F\left(x,y\right)=-k_{B}T\;\log\left[P\left(x,y\right)\right]$$

where $k_{B}$ is the Boltzmann constant, T is the absolute temperature, and P(x,y) is the probability of x for *d_1(i)_*[O2_(i)_–O3_(i+1)_] and y for *d_2(i)_*[O4_(i)_–O4_(i+1)_] distances.

Next, the flips of glucose subunits for various conformations were monitored by angle between a pair of adjacent glucose units, *θ_(i)_*[C6_(i)_–C2_(i+1)_–C6_(i+1)_], as defined in Figure 3a. Without the flipping, the C6 atoms are in the same site with the adjacent unit. Thus, the *θ_(i)_*[C6_(i)_–C2_(i+1)_–C6_(i+1)_] values in no-flip structures are less than 90 degrees. In contrast, when the unit was flipped, the C6 atoms were opposite to those of the adjacent unit, and its *θ_(i)_*[C6_(i)_–C2_(i+1)_–C6_(i+1)_] was higher than 90 degrees. In addition, the radius of gyration ($R_{g}$) was calculated. The $R_{g}$ represents the mass-weighted scalar length of each atom from the center of gravity of the molecule, calculated using Equation (2).

(2) $$R_{g}=\sqrt{\frac{\sum_{i=1}^{N}m_{i}{\left(r_{i}-r_{cm}\right)}^{2}}{\sum_{i=1}^{N}m_{i}}}$$

where N is the number of atoms, $m_{i}$ is the mass of atom i, $r_{i}$ is the Cartesian position vector of atom i, and $r_{cm}$ is the center of mass of CD.

The next parameter is a modified circularity (C), which is a dimensionless shape factor based on our previous work [34]. Here, we measured the diameter *d_3(i)_*[C_g(Glu(i))_–O4_(i+3)_], which is a distance between the center of mass of the *i*-th glucose unit (C_g(Glu(i))_) and the glycosidic oxygen atom of the opposite glucose unit (O4_(i+3)_), as depicted in Figure 3b. There are seven *d_3(i)_* parameters for each HPβCDs snapshot, since there are seven glucose subunits in βCDs. Hence, C is defined as:

(3) $$C=\frac{\underset{i\in \left\{1\ldots 7\right\}}{\min}\left(d_{3\left(i\right)}\right)}{\underset{i\in \left\{1\ldots 7\right\}}{\max}\left(d_{3\left(i\right)}\right)}$$

Note that the value of C is in a range of 0 < C ≤ 1. If C is equal to 1.0, where min(*d_3(i)_*) = max(*d_3(i)_*), it means that the cavity has a perfectly circular shape. However, when C becomes deviated from 1.0, this is an indication for conformational changes to an elliptical shape. Finally, in order to study the cavity self-closure of HPβCDs, we define the *d_4(i)_*[C_g(βCD)_–C_g(HP(i))_] parameter, which is the distance of the center of mass between the βCD ring and each HP group, as demonstrated in Figure 3c. All parameters were analyzed from 25,000 snapshots taken from REMD simulations. The analyzed results at 300 K will be presented and discussed in the next section, while those of other temperatures are provided in the Supplementary Materials.

## 3. Results and Discussion

### 3.1. Structural Analysis

#### 3.1.1. Structural Distortion of Glucose Units in the HPβCDs

**Figure 4:**
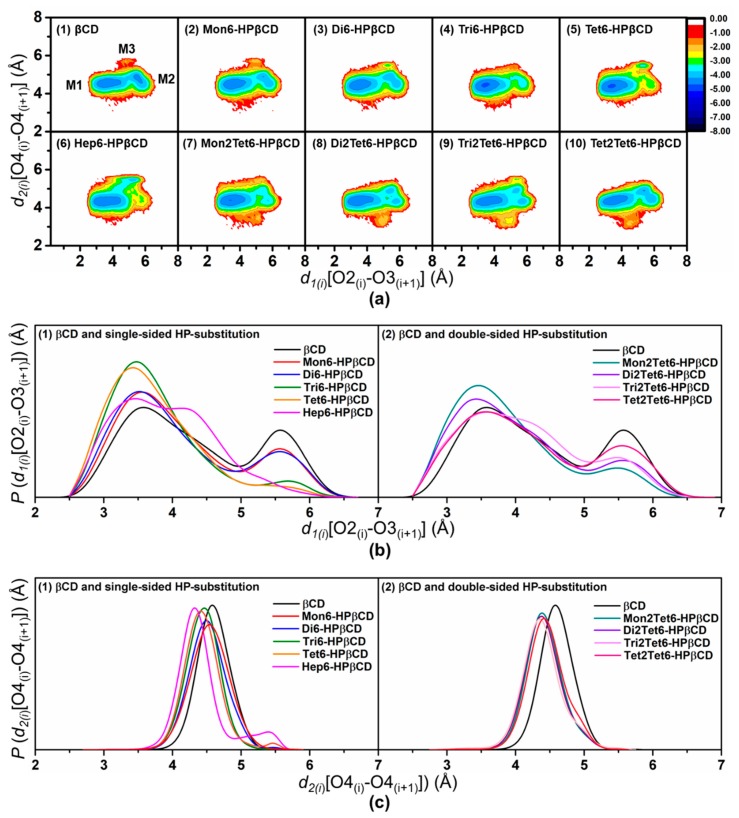
(**a**) Contour graphs of the probability distributions in terms of free energy, (**b**) the probability of *d_1(i)_*, and (**c**) the probability of *d_2(i)_* from a total of 25,000 snapshots at 300 K of REMD simulations with the glycam06 force field for βCD and HPβCDs.

To investigate the conformational changes of βCD and all selected HPβCDs, the probability distributions of *d_1(i)_*[O2_(i)_–O3_(i+1)_] and *d_2(i)_*[O4_(i)_–O4_(i+1)_] distances for all models at 300 K were calculated in terms of free energy, using Equation (1), and these are plotted in Figure 4a. The contour graphs give the values of the distance probability, where the denoted darkest blue color (ranked from dark red to blue) represents the lowest free energy.

In the case of βCD, three conformational minima (M1, M2, and M3) were detected as shown in Figure 4(a1), which is in agreement with previous results from MD in aqueous solution [29]. The most likely distribution at M1 was found with the *d_1(i)_* and *d_2(i)_* distances at around 3.5 and 4.5 Å, respectively, describing the almost perfect conical shape of the βCD ring. The second and third main populations were M2 and M3, with *d_1(i)_*/*d_2(i)_* of around 5.5 Å/4.5 Å and 5.0 Å/5.5 Å, respectively. When comparing βCD with a large-ring CD (εCD) by the REMD method [33], three important minima were found to be similar to the βCD results, but the M3 population of εCD was higher than that of βCD, due to the large size and the high flexibility of the macroscopic ring, having a higher probability of flipping the glucopyranose units in the large-ring system.

From all of the free energy plots of HPβCDs with single- and double-sided HP-substitutions, the *d_2(i)_*[O4_(i)_–O4_(i+1)_] values fluctuated in the range of 4.0–5.0 Å, implying that the backbone structures of HPβCD were not greatly affected by the HP-substitution groups. In contrast, it was noticeable that *d_1(i)_*[O2_(i)_–O3_(i+1)_] was in a wide range around 2.5–6.5 Å. Therefore, we plotted the probability of *d_1(i)_*[O2_(i)_–O3_(i+1)_] and *d_2(i)_*[O4_(i)_–O4_(i+1)_], as depicted in Figure 4b,c, respectively, in order to compare the conformational minima among the models. For HPβCDs with single-sided HP-substitutions, the most dominant population of *d_1(i)_*[O2_(i)_–O3_(i+1)_] for the Tri6- and Tet6-HPβCDs was around 3.5 Å, indicating the narrow behavior of their secondary rim. Additionally, the conformational changes of other models were quite similar to the native βCD, except for Hep6-HPβCD with *d_1(i)_*[O2_(i)_–O3_(i+1)_] at around 4.0-5.0 Å. Consequently, M3 was found in Hep6-HPβCD higher than βCDs and the other 6-HPβCDs, resulting from the steric hindrance of seven HP groups at the primary rim.

In the case of HPβCDs with double-sided HP-substitution, the conformational changes were quite similar to the βCD, in which three main probability distributions were found, following this order: M1 > M2 > M3. When increasing the number of HP at the O2 positions, the M1 population decreased, while the M2 population increased, as depicted in Figure 4(a7–a10), which were related to the distribution of *d_1(i)_*[O2_(i)_–O3_(i+1)_] (Figure 4(b2)). In addition, the new probability distribution with *d_1(i)_*/*d_2(i)_* around 4.3 Å/3.5 Å was observed when adding a HP group on the secondary rim, and this probability distribution was enhanced when increasing the number of HP at the O2 positions.

The results indicate that the substitution of the HP groups on narrow and/or wider rims directly affected the distortion in the βCD ring. It is a worth noting that the M1 population was dramatically increased, whilst M2 was decreased and M3 completely disappeared due to complexation with low-water-soluble compounds [35,36,37].

#### 3.1.2. Flipping of the HPβCDs

**Figure 5:**
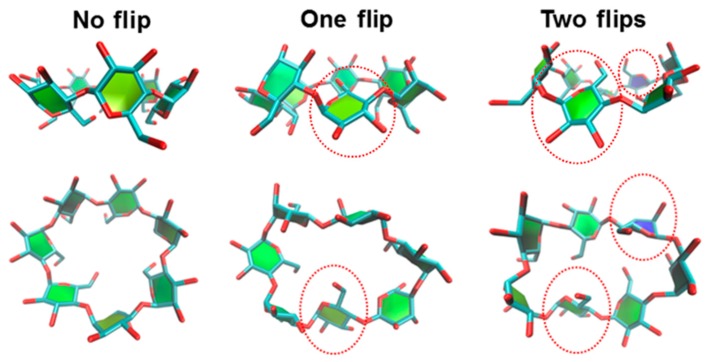
The flipped conformations of βCD with different numbers of flip glucose subunits, which were selected from the simulation at 300 K.

It is noticeable that the flips of glucose subunits were related directly to the distortion of the CD structure. As mention earlier, a flip of the glucose units was counted when *θ_(i)_*[C6_(i)_–C2_(i+1)_–C6_(i+1)_] was higher than 90 degrees. According to the *θ_(i)_*[C6_(i)_–C2_(i+1)_–C6_(i+1)_] analysis, the results can be divided into three main different flip-conformations: which are (1) no flip; (2) one flip; (3) two flips. Example snapshots of flipped βCD conformations are shown in Figure 5.

The probabilities of different numbers of flip glucose subunits for all models at 300 K are summarized in Table 2. The no-flip conformation was the main population in all models, while other higher flips (one- and two-flip) were found to be of minor importance during the simulation. In all HPβCDs, more than 58% of no-flip conformations were observed, whereas βCD showed higher values of one- and two-flip conformations. The highest percentages of one- and two-flip conformations in βCD corresponded to the long length of *d_1(i)_*[O2_(i)_–O3_(i+1)_] detected around 5.0–6.0 Å in the M2 population, as shown in Figure 4(a1). With an increasing number of HP only at O6 positions of glucose subunits, the no-flip population increased by up to 75–78% in Tri6- and Tet6-HPβCDs. Meanwhile, the one-flip population decreased from 35% to 21%. These results were related to the large population of *d_1(i)_*[O2_(i)_–O3_(i+1)_] at 3.5 Å for Tri6- and Tet6-HPβCDs. For HPβCDs with double-sided HP-substitution, the no-flip population also increased when compared with those of native βCD. However, the one- and two-flip populations of these of double-sided HP-substitutions were higher than those of Tet6-HPβCDs. In addition, the trends of these flips were not significantly different at other temperatures, as shown in Table S1. The percentage of no-flip angles correlates with M1, which is the most populated state during simulation, and the percentages of flip angles relate with M2 and M3, in which the glucopyranose subunits flip and the intramolecular hydrogen bonds of the wider CD rim disappear. Thus, glucose subunits in both 6-HPβCDs and 2,6-HPβCDs have a lower chance to flip (22–31% of the flip angle) than in βCD (42% of flip angle), which is in agreement with their inclusion efficiency reported in our previous study [22].

#### 3.1.3. Radius of Gyration

**Figure 6:**
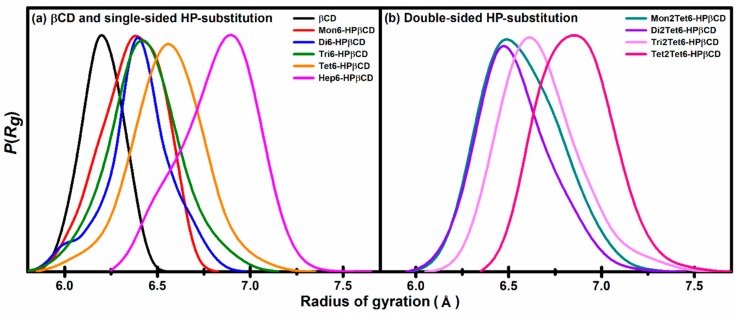
The probability of the radius of gyration for βCD and all HPβCDs at 300 K.

The flipping of the glucose subunits directly influences the distortion of the macrocyclic ring, as shown in Figure 5 (top view of the different flipped conformations). Therefore, the shapes of all models were investigated in terms of the radius of gyration ($R_{g}$), and the results are shown in Figure 6. The average of $R_{g}$ of native βCD, Mon6-, Di6-, Tri6-, Tet6-, and Hep6-HPβCDs are 6.18, 6.38, 6.39, 6.45, 6.55, and 6.85 Å, respectively, and those of Mon2Tet6-, Di2Tet6-, Tri2Tet6-, and Tet2Tet6-HPβCDs are 6.45, 6.46, 6.65, and 6.85 Å. The results show that the average $R_{g}$ trends to increase when adding more HP groups, which are similar to the $R_{g}$ results from a molecular dynamics simulation, as reported by Yong et al. [17]. However, for Hep6-HPβCD and Tri2Tet6-HPβCD with the same degree of substitution (1.00), the average $R_{g}$ of Hep6-HPβCD was higher than that of Tri2Tet6-HPβCD, because of the higher steric hindrance of the seven HP groups at the primary rim of Hep6-HPβCD. Overall, the $R_{g}$ in the substituted models was observed to be higher than the native βCD, because of the fluctuation and steric hindrance of the HP groups.

#### 3.1.4. Circularity

To determine the effect of the HP-substitutions on the geometry of the CD cavity, we used the circularity (C) calculated following Equation (3). The C values of all models at 300 K are listed in Table 3. βCD had the lowest C (0.727 ± 0.087), indicating that its conformational change is higher than those of other models. Moreover, the C values of Tet2Tet6-HPβCDs are also very low (0.742 ± 0.080). The reason for this more flexible conformation may be the result from the higher steric hindrance of HP groups on both the O2 and O6 positions of the glucose subunits. In addition, taking the standard deviation into account, the C values of Tri6- and Tet6-HPβCDs were found to be 0.814 ± 0.075 and 0.815 ± 0.076, respectively. The data suggests that the cavity becomes more circular in shape, when increasing the number of substituting the HP at the O6 primary hydroxyl groups of up to three or four residues, corresponding to a high population of no-flip conformations. Although the population of no-flip conformations of Tri6-, Tet6- and Mon2Tet6-HPβCDs, as well as that of Di2Tet6-HPβCD, are quite similar (higher than 75%), the C values of Mon2Tet6- and Di2Tet6-HPβCDs are lower than the Tri6- and Tet6-HPβCDs, due to the steric hindrance of the HP groups, leading to a distortion of some glucose subunits in the no-flip conformation. For that reason, Tri6- and Tet6-HPβCDs are highlighted as proper structures for forming inclusion complexes with guest molecules, as indicated from their high circularities with a lower possibility of flipping.

### 3.2. Cavity Self-Closure

**Figure 7:**
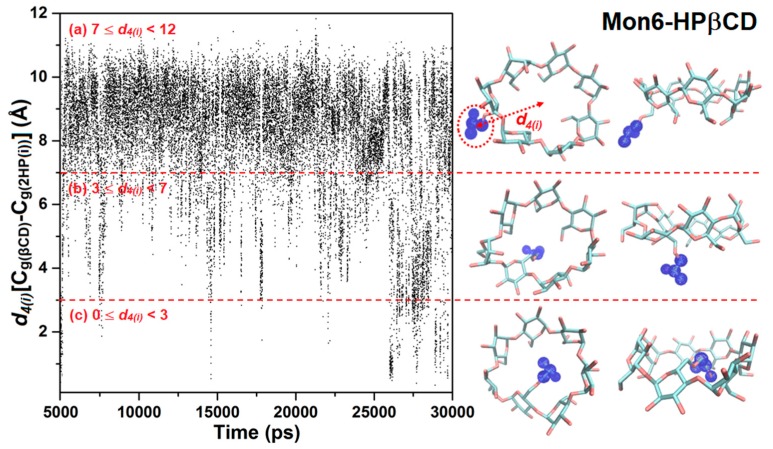
The distance of the centers of mass between the βCD ring and HP group, *d_4(i)_*[C_g(βCD)_–C_g(HP(i))_] for Mon6-HPβCD at 300 K.

The arrangement of the HP groups during simulations influences the CD cavity accessibility directly. Some HP groups can point toward the CD interior, leading to self-closure of the CD cavity. The arrangements of the HP substituents at the glucose subunits were monitored via the distance of the center of mass between the βCD ring and each HP group, called *d_4(i)_*[C_g(βCD)_–C_g(HP(i))_]. The arrangement of HP for Mon6-HPβCD is plotted in Figure 7, while those of the other HPβCDs are shown in Figure S2.

**Figure 8:**
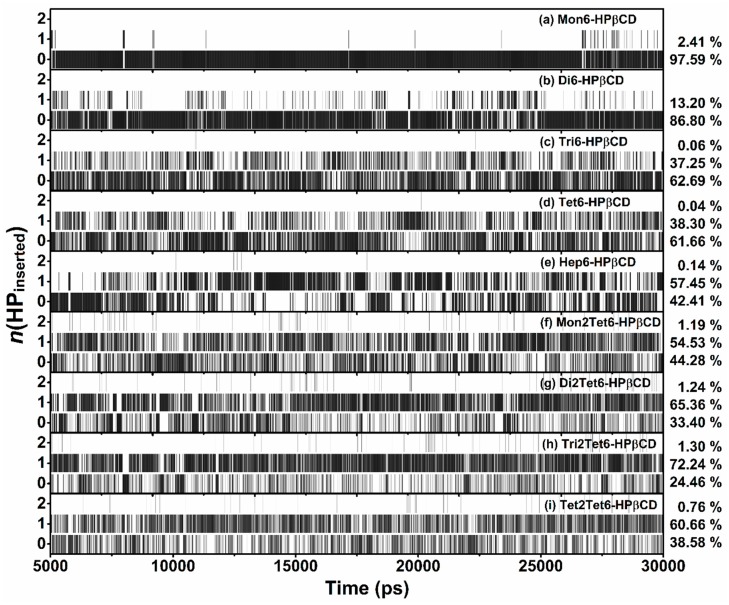
The probability of *n*(HP_inserted_) from 25,000 snapshots (criteria: *d_4(i)_*[C_g(βCD)_–C_g(HP(i))_] < 3 Å) at 300 K for all HPβCDs.

During the REMD simulation of Mon6-HPβCD, almost 98% of HP groups pointed toward the CD exterior, with *d_4(i)_*[C_g(βCD)_–C_g(HP(i))_] around 7–10 Å. For Mon6-HPβCD with *d_4(i)_*[C_g(βCD)_–C_g(HP(i))_] in the rank from 7 to 12 Å, the shape of Mon6-HPβCD seemed to be like a bowl (Figure 7a). When the *d_4(i)_*[C_g(βCD)_–C_g(HP(i))_] was less than 3 Å, the HP group was hindered and rotated into the cavity of the βCD ring, leading to self-closure of the CD cavity (Figure 7c). We also found that the cavity self-closure was related to flipping of the HP-substituted glucose subunits, which triggers HP entrance into the CD cavity (*d_4(i)_*[C_g(βCD)_–C_g(HP(i))_] ~ 1 Å). Some snapshots show that the substituents enter the cavity at the narrow rim with *d_4(i)_*[C_g(βCD)_–C_g(HP(i))_] ~ 2 Å. For that reason, we defined the self-closure of CD cavity or HP occupied in the CD cavity when *d_4(i)_*[C_g(βCD)_–C_g(HP(i))_] < 3 Å. The numbers of *d_4(i)_*[C_g(βCD)_–C_g(HP(i))_] < 3 Å were called *n*(HP_inserted_), as illustrated in Equation (4). For all REMD simulations of HPβCDs at 300 K, *n*(HP_inserted_) are plotted in Figure 8 with the probability in terms of the percentage of 25,000 snapshots. The results at the various temperatures are summarized in Table S2.

*n*(HP_inserted_) = *n*(*d_4(i)_*[C_g(βCD)_–C_g(HP(i))_] < 3 Å) (4)

**Figure 9:**
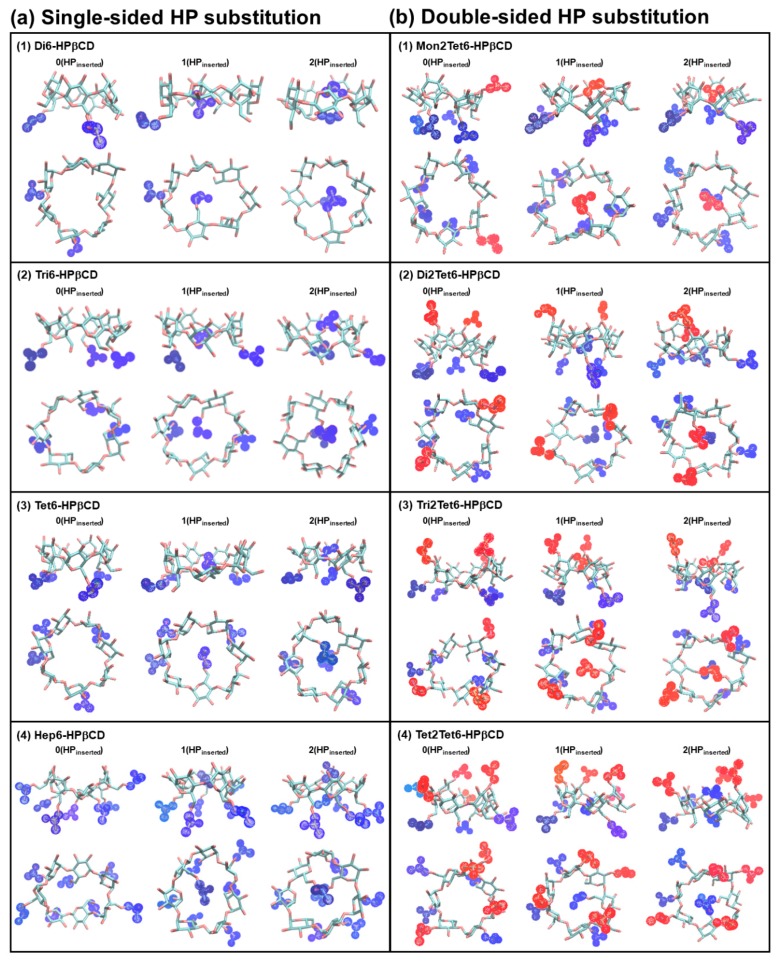
Example snapshots of different numbers of HP occupied in the CD cavity (criteria: *d_4(i)_*[C_g(βCD)_–C_g(HP(i))_] < 3 Å) for all HPβCDs at 300 K.

For HPβCDs with single-sided HP-substitutions, it was noticeable that when *n*(HP_inserted_) > 0, the cavity self-closure occurred. Example snapshots of HPβCDs with different numbers of *n*(HP_inserted_) are given in Figure 9. As shown in the figure, the flipped HP-substituted glucopyranose subunits and the HP groups inserted into the hydrophobic cavity led to cavity self-closure in the HPβCDs. When the number of HP groups was increased, the percentage of cavity self-closure significantly increased from 2.41 to 57.59% in Hep6-HPβCD. This indicates that the cavity of the HPβCD structure was almost blocked when the number of HP-substitutions was increased. Furthermore, the maximum of *n*(HP_inserted_) was equal to 2, even in the HPβCD with a high degree of HP-substitutions, such as Hep6-HPβCD and Tet2Tet6-HPβCD.

For HPβCDs with double-sided HP-substitutions, the HP groups, both on the narrow and wider rim of HPβCD could point into the cavity. For that reason, the percentage of cavity self-closure was higher than those of single-sided HP-substitutions, because of the large fluctuation of their structures. When increasing the number of HP groups at the O2 positions on Tet6-HPβCD from one to four, the probability of cavity self-closure rose dramatically from 38.34% (Tet6-HPβCD) to 55.72, 66.60, 73.54, and 61.42% for Mon2Tet6-, Di2Tet6-, Tri2Tet6-, and Tet2Tet6-HPβCDs, respectively. With increasing temperatures, the glucose subunits distorted, and the HP groups fluctuated far from the center of mass of the βCD ring, leading to a lower probability of cavity self-closure at higher temperatures for all models (Table S2).

In summary, all data showed that the HP-substitution strongly affects the cavity self-closure of HPβCD, especially when the number of the HP substituents increases. To prevent cavity self-closure, which might block the inclusion of the guest molecules, we suggest that the HP-substitution should be a single-sided substitution, with the degree of substitution being less than 0.57 such as Di-, Tri-, and Tet6-HPβCDs. However, it should be mentioned that the above results were only valid in cases where there was no interaction between the cyclodextrins (at higher concentrations). Moreover, the change of the dielectric properties, e.g., by using solvent mixtures, might influence the conformations of the considered molecules, as well as their conformational equilibria.

## 4. Conclusions

REMD simulations have been performed on HPβCDs with various degrees of substitution (DS = 0.14–1.14) to study the structural behaviors and the effects of HP substituents. Several parameters that influence such changes have been identified. The circularity and the radius of gyration explain the size and shape of the cavity of the ring, and the flip angle and important distances describe the conformational changes and the flexibilities of the HP groups. The results show that HPβCDs have a more pronounced conical shape than βCD; however, cavity self-closure occurs because some glucopyranoses with HP groups flip or distort, followed by one or two HP groups coming close to the CD cavity, thus hindering drug inclusion. HPβCDs with high DS are more likely to be blocked, while HPβCDs with double-sided HP-substitutions are even more likely to be blocked. Among the nine HPβCDs, all analysis parameters point out that Tri- and Tet6-HPβCDs with three and four HP-substitutions on the primary rim have a distinctive conformation, being mostly circular with a low possibility of flipping and cavity enclosure. Thus, these HPβCDs could serve as more proper hosts for the encapsulation of low-water soluble compounds.

## Figures and Tables

**Table 1:** Model summary of introducing HP groups at the O2 and/or O6 positions on glucose subunits.

Models	Degree of Substitution	O2 Substitution	O6 Substitution
βCD	0.00	None	None
**Single-sided HP-substitution**			
Mon6-HPβCD	0.14	None	1 (At glucose unit 1)
Di6-HPβCD	0.28	None	2 (At glucose units 1 and 3)
Tri6-HPβCD	0.43	None	3 (At glucose units 1, 3, and 5)
Tet6-HPβCD	0.57	None	4 (At glucose units 1, 3, 5, and 7)
Hep6-HPβCD	1.00	None	7 (At all glucose units)
**Double-sided HP-substitution**			
Mon2Tet6-HPβCD	0.71	1 (At glucose unit 4)	4 (At glucose units 1, 3, 5, and 7)
Di2Tet6-HPβCD	0.85	2 (At glucose units 2 and 6)	4 (At glucose units 1, 3, 5, and 7)
Tri2Tet6-HPβCD	1.00	3 (At glucose units 2, 4, and 6)	4 (At glucose units 1, 3, 5, and 7)
Tet2Tet6-HPβCD	1.14	4 (At glucose units 2, 4, 6, and 7)	4 (At glucose units 1, 3, 5, and 7)

**Table 2:** The probability of different numbers of flip glucose subunits in βCD and HPβCDs, using the flip angle parameter, *θ_(i)_*[C6_(i)_–C2_(i+1)_–C6_(i+1)_] at 300K (criteria: values higher than 90 degrees).

Models	The Percentage of the Flip Angle (%)		
	No Flip	One Flip	Two Flips
βCD	58	35	7
**Single-sided HP-substitution**			
Mon6-HPβCD	69	28	3
Di6-HPβCD	74	23	3
Tri6-HPβCD	75	24	1
Tet6-HPβCD	78	21	1
Hep6-HPβCD	73	25	2
**Double-sided HP-substitution**			
Mon2Tet6-HPβCD	77	22	1
Di2Tet6-HPβCD	75	24	1
Tri2Tet6-HPβCD	70	28	2
Tet2Tet6-HPβCD	74	24	2

**Table 3:** The average and standard deviation of the circularity (C) of βCD and HPβCDs at 300 K, using REMD simulations.

**Models**	C
βCD	0.727 ± 0.087
**Single-sided HP-substitution**	
Mon6-HPβCD	0.746 ± 0.085
Di6-HPβCD	0.751 ± 0.086
Tri6-HPβCD	0.814 ± 0.075
Tet6-HPβCD	0.815 ± 0.076
Hep6-HPβCD	0.773 ± 0.088
**Double-sided HP-substitution**	
Mon2Tet6-HPβCD	0.803 ± 0.075
Di2Tet6-HPβCD	0.767 ± 0.076
Tri2Tet6-HPβCD	0.785 ± 0.074
Tet2Tet6-HPβCD	0.742 ± 0.080

## Supplementary Materials

The following are available online at http://www.mdpi.com/2073-4360/11/1/145/s1, Figure S1: The overlapping between the potential energy distributions of each replica temperature, ranging from 269.5 K to 570.9 K. Figure S2: The distance of the centers of mass between the βCD ring and the HP group, *d_4(i)_*[C_g(βCD)_–Cg_(HP(i))_] for (a) Di6-HPβCD, (b) Tri6-HPβCD, (c) Tet6-HPβCD, (d) Hep6-HPβCD, (e) Mon2Tet6-HPβCD, (f) Di2Tet6-HPβCD, (g) Tri2Tet6-HPβCD, and (h) Tet2Tet6-HPβCD at 300 K. Figures S3–S12: Contour graphs of the native βCD (S3), Mon6-HPβCD (S4), Di6-HPβCD (S5), Tri6-HPβCD (S6), Tet6-HPβCD (S7), Hep6-HPβCD (S8), Mon2Tet6-HPβCD (S9), Di2Tet6-HPβCD (S10), Tri2Tet6-HPβCD (S11), and Tet2Tet6-HPβCD (S12), probability distributions of 25,000 snapshots with the glycam06 force field at various temperatures. Figure S13: The radius of gyration at various temperatures for (a) βCD, (b) Mon6-HPβCD, (c) Di6-HPβCD, (d) Tri6-HPβCD, (e) Tet6-HPβCD, (f) Hep6-HPBCD, (g) Mon2Tet6-HPBCD, (h) Di2Tet6-HPBCD, (i) Tri2Tet6-HPBCD, and (j) Tet2Tet6-HPBCD. Table S1: The probabilities of different numbers of flip glucose subunits in βCD and all HPβCDs, using the flip angle parameter, *θ_(i)_*[C6_(i)_–C2_(i+1)_–C6_(i+1)_] at various temperatures (criteria: a value higher than 90 degree) compared with the classical MD simulation in the parenthesis. Table S2: The probability of the overall snapshots with different numbers of HP occupied in the CD cavity (criteria: *d_4(i)_*[C_g(βCD)_–Cg_(HP(i))_] < 3 Å) for all HPβCDs at various temperatures.
