# Supplementary material for: Cavity Closure of 2-Hydroxypropyl-β-Cyclodextrin: Replica Exchange Molecular Dynamics Simulations
Source: Polymers (Basel). 2019 Jan 16;11(1):145. doi: 10.3390/polym11010145 (PMC6401915; doi:10.3390/polym11010145)
Supplement: Supplementary file 1 [file polymers-11-00145-s001.pdf]

Supplementary Materials

Cavity Closure of 2-Hydroxypropyl-β-Cyclodextrin: Replica Exchange Molecular Dynamics Simulations

Khanittha Kerdpol [1], Jintawee Kicuntod [2], Peter Wolschann [2,3,4], Seiji Mori [5], Chompoonut Rungnim [6], Manaschai Kunaseth [6], Hisashi Okumura [7], Nawee Kungwan [1,8,*] and Thanyada Rungrotmongkol [2,9,10,*]

1 Department of Chemistry, Faculty of Science, Chiang Mai University, Chiang Mai 50200, Thailand; khanittha.view@gmail.com
2 Structural and Computational Biology Research Unit, Department of Biochemistry, Faculty of Science, Chulalongkorn University, Bangkok 10330, Thailand; jintawee.ki@gmail.com (J.K.); karl.peter.wolschann@univie.ac.at (P.W.)
3 Department of Pharmaceutical Chemistry, University of Vienna, Vienna 1090, Austria
4 Institute of Theoretical Chemistry, University of Vienna, Vienna 1090, Austria
5 Institute of Quantum Beam Science, Graduate School of Science and Engineering, Ibaraki University, 2-1-1 Bunkyo, Mito, Ibaraki 310-8512, Japan; seiji.mori.compchem@vc.ibaraki.ac.jp
6 National Nanotechnology Center (NANOTEC), National Science and Technology Development Agency (NSTDA), Pathum Thani 12120, Thailand; chompoonut@nanotec.or.th (C.R.); manaschai@nanotec.or.th (M.K.)
7 Research Center for Computational Science, Institute for Molecular Science, Okazaki, Aichi 444-8585, Japan; hokumura@ims.ac.jp
8 Center of Excellence in Materials Science and Technology, Chiang Mai University, Chiang Mai 50200, Thailand
9 Ph.D. Program in Bioinformatics and Computational Biology, Faculty of Science, Chulalongkorn University, Bangkok 10330, Thailand
10 Molecular Sensory Science Center, Faculty of Science, Chulalongkorn University, 254 Phayathai Road, Patumwan, Bangkok 10330, Thailand
* Correspondence: naweekung@gmail.com (N.K.); thanyada.r@chula.ac.th (T.R.); Tel.: +66-5394-3341 (ext. 101) (N.K.); +66-2218-5426 (T.R.)

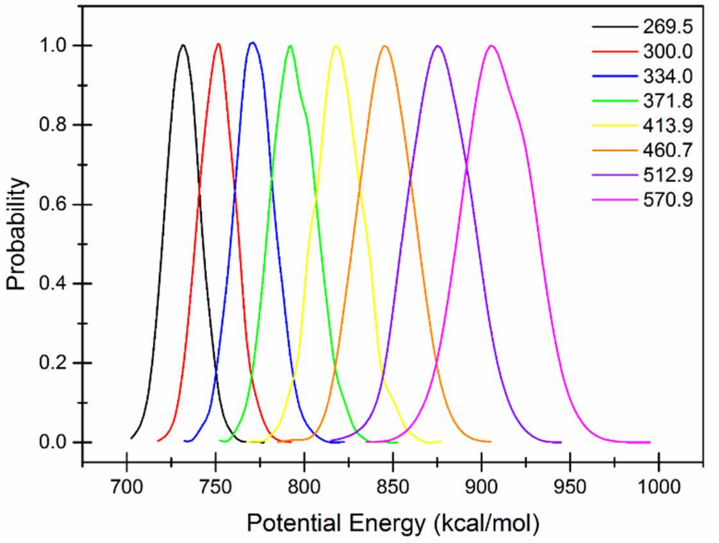

Figure S1. The overlapping between the potential energy distributions of each replica temperature ranging from 269.5 K to 570.9 K.

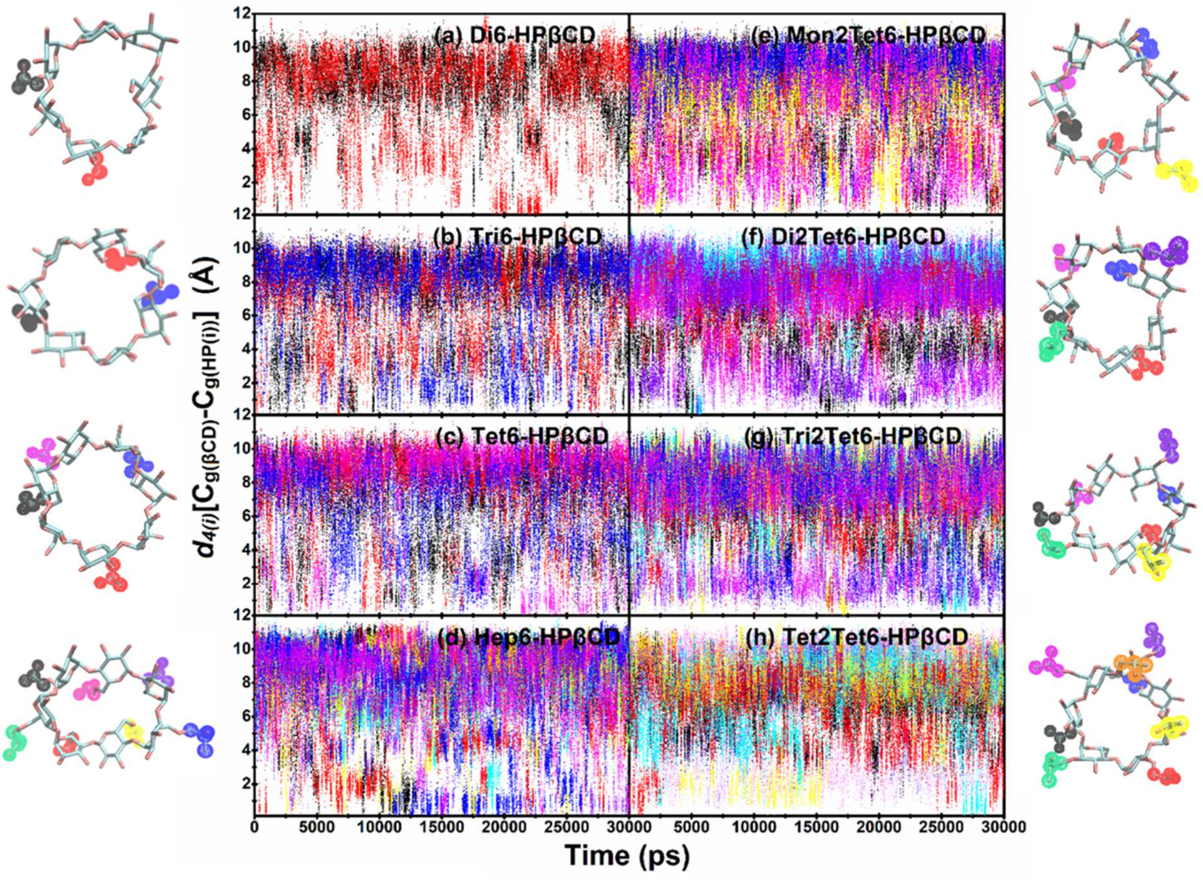

Figure S2. The distance of centers of mass between βCD ring and HP group, $d_{4(i)}[C_{g(\beta CD)}–C_{g(HP(i))}]$ for (**a**) Di6-HPβCD, (**b**) Tri6-HPβCD, (**c**) Tet6-HPβCD, (**d**) Hep6-HPβCD, (**e**) Mon2Tet6-HPβCD, (**f**) Di2Tet6-HPβCD, (**g**) Tri2Tet6-HPβCD, and (**h**) Tet2Tet6-HPβCD at 300 K.

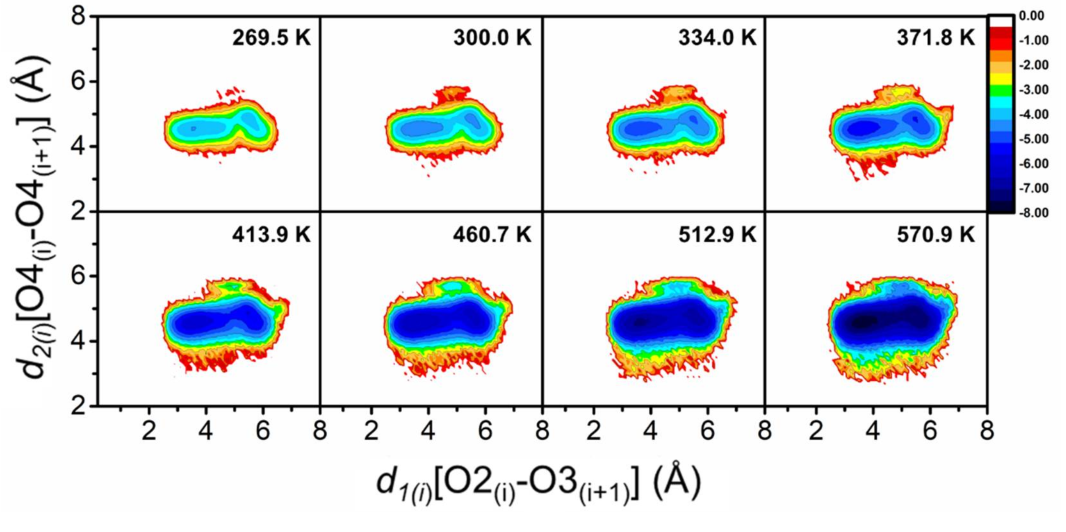

Figure S3. Contour graphs of the native βCD probability distribution of 25,000 snapshots with the glycam06 force field at various temperatures.

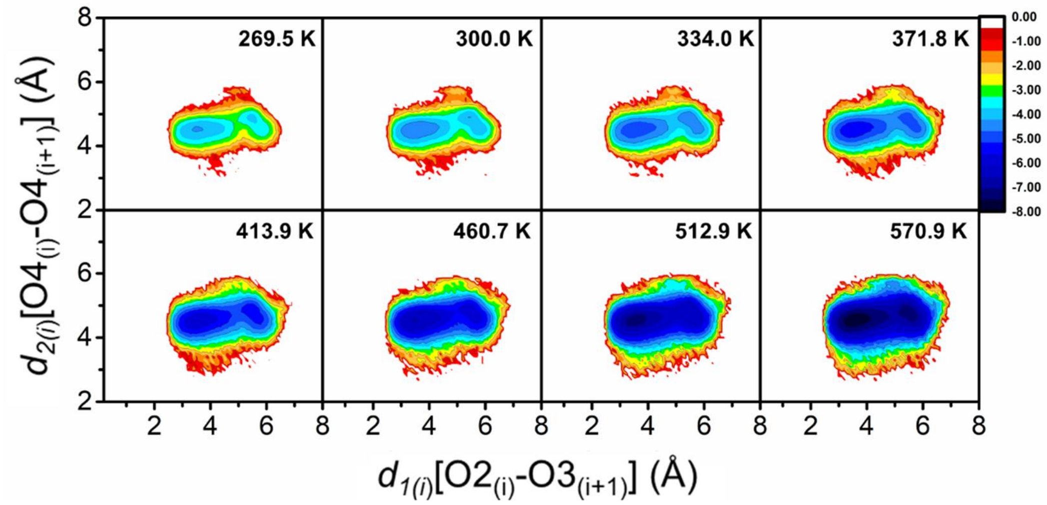

Figure S4. Contour graphs of the probability distribution of 25,000 snapshots of Mon6-HPβCD with the glycam06 force field in the various temperatures.

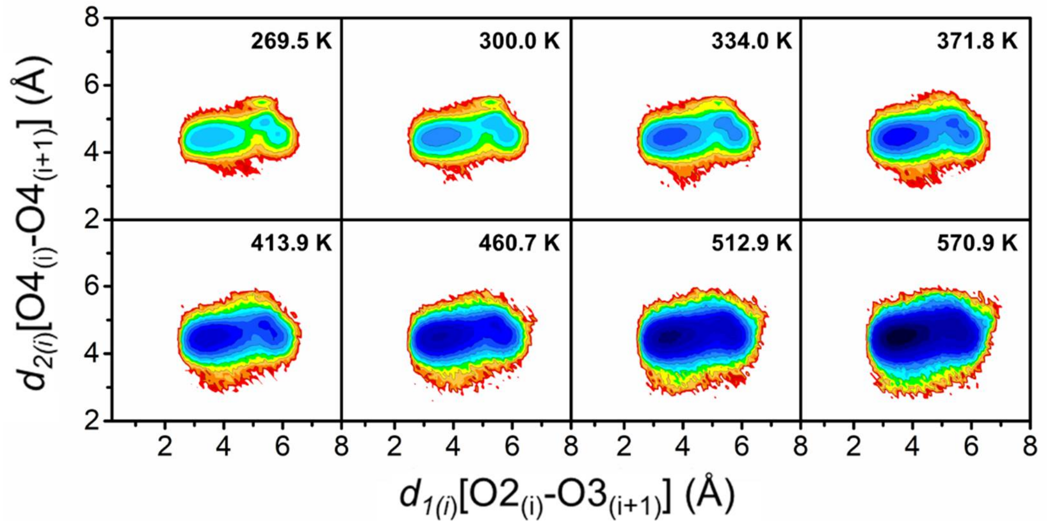

Figure S5. Contour graphs of the probability distribution of 25,000 snapshots of Di6-HPβCD with the glycam06 force field in the various temperatures.

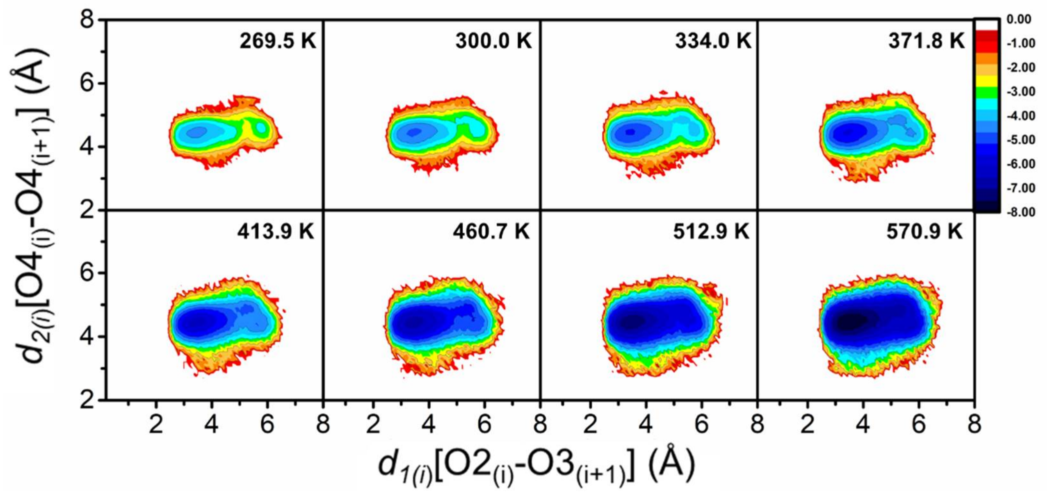

Figure S6. Contour graphs of the probability distribution of 25,000 snapshots of Tri6-HPβCD with the glycam06 force field in the various temperatures.

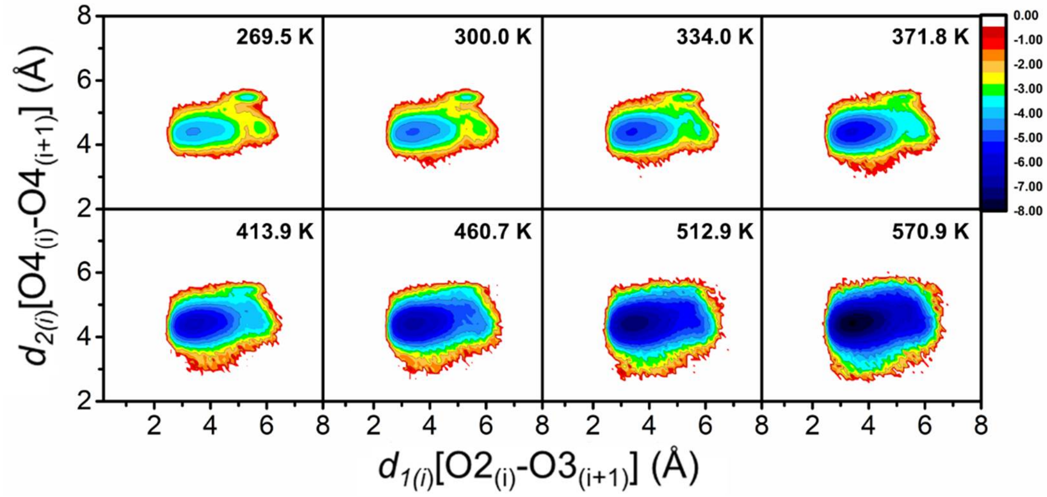

Figure S7. Contour graphs of the probability distribution of 25,000 snapshots of Tet6-HPβCD with the glycam06 force field in the various temperatures.

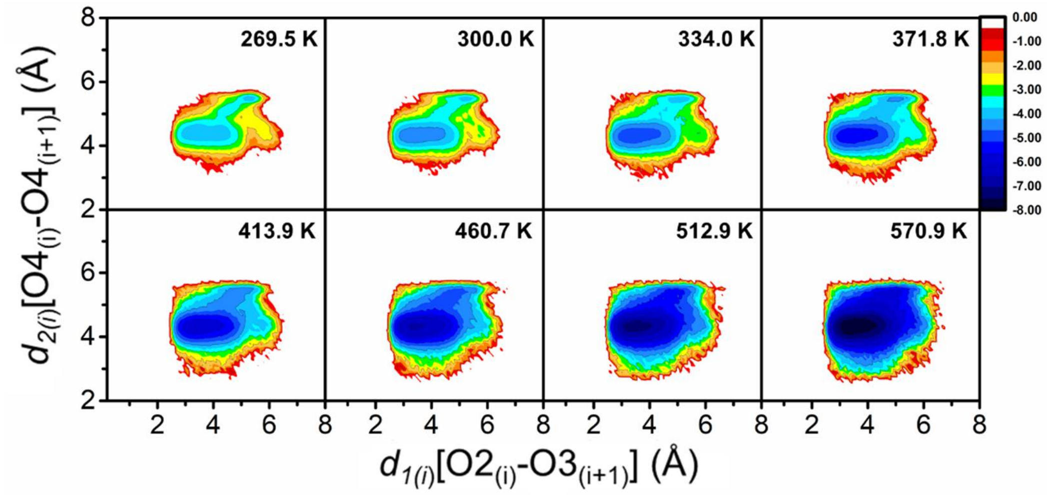

Figure S8. Contour graphs of the probability distribution of 25,000 snapshots of Hep6-HPβCD with the glycam06 force field in the various temperatures.

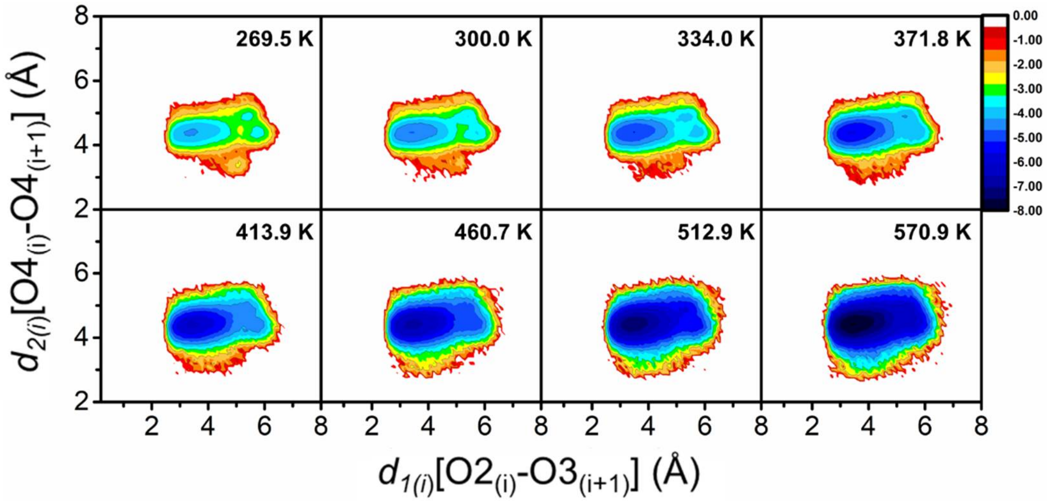

Figure S9. Contour graphs of the probability distribution of 25,000 snapshots of Mon2Tet6-HPβCD with the glycam06 force field in the various temperatures.

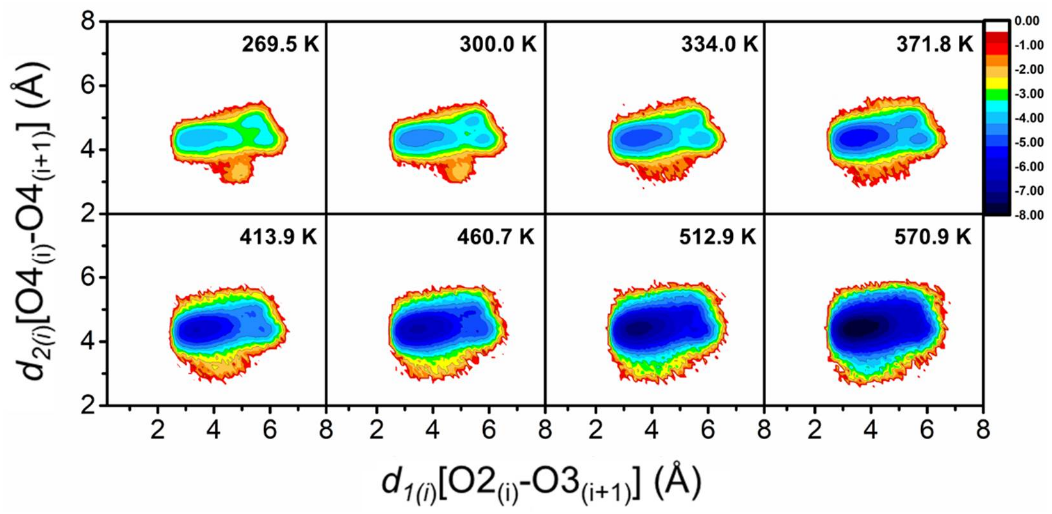

Figure S10. Contour graphs of the probability distribution of 25,000 snapshots of Di2Tet6-HPβCD with the glycam06 force field in the various temperatures.

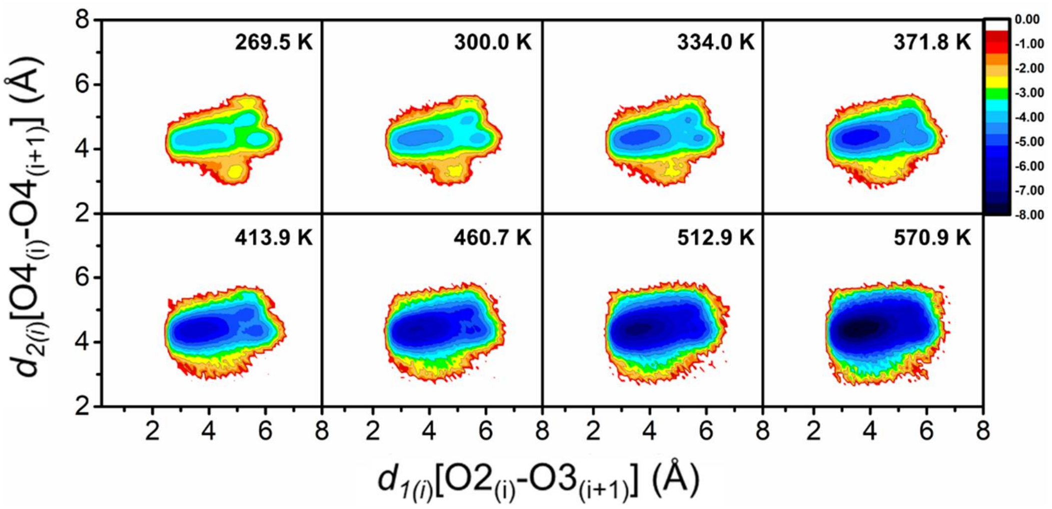

Figure S11. Contour graphs of the probability distribution of 25,000 snapshots of Tri2Tet6-HPβCD with the glycam06 force field in the various temperatures.

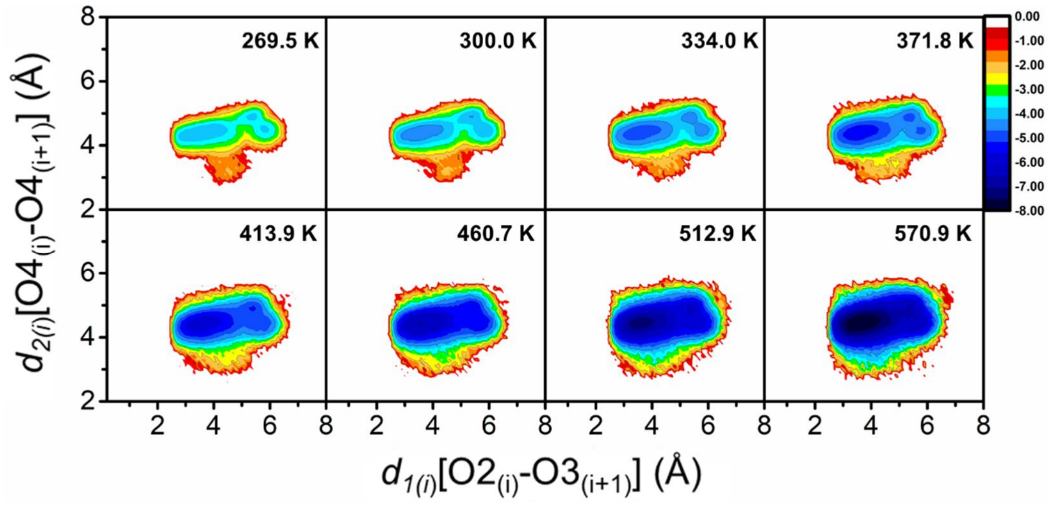

Figure S12. Contour graphs of the probability distribution of 25,000 snapshots of Tet2Tet6-HPβCD with the glycam06 force field in the various temperatures.

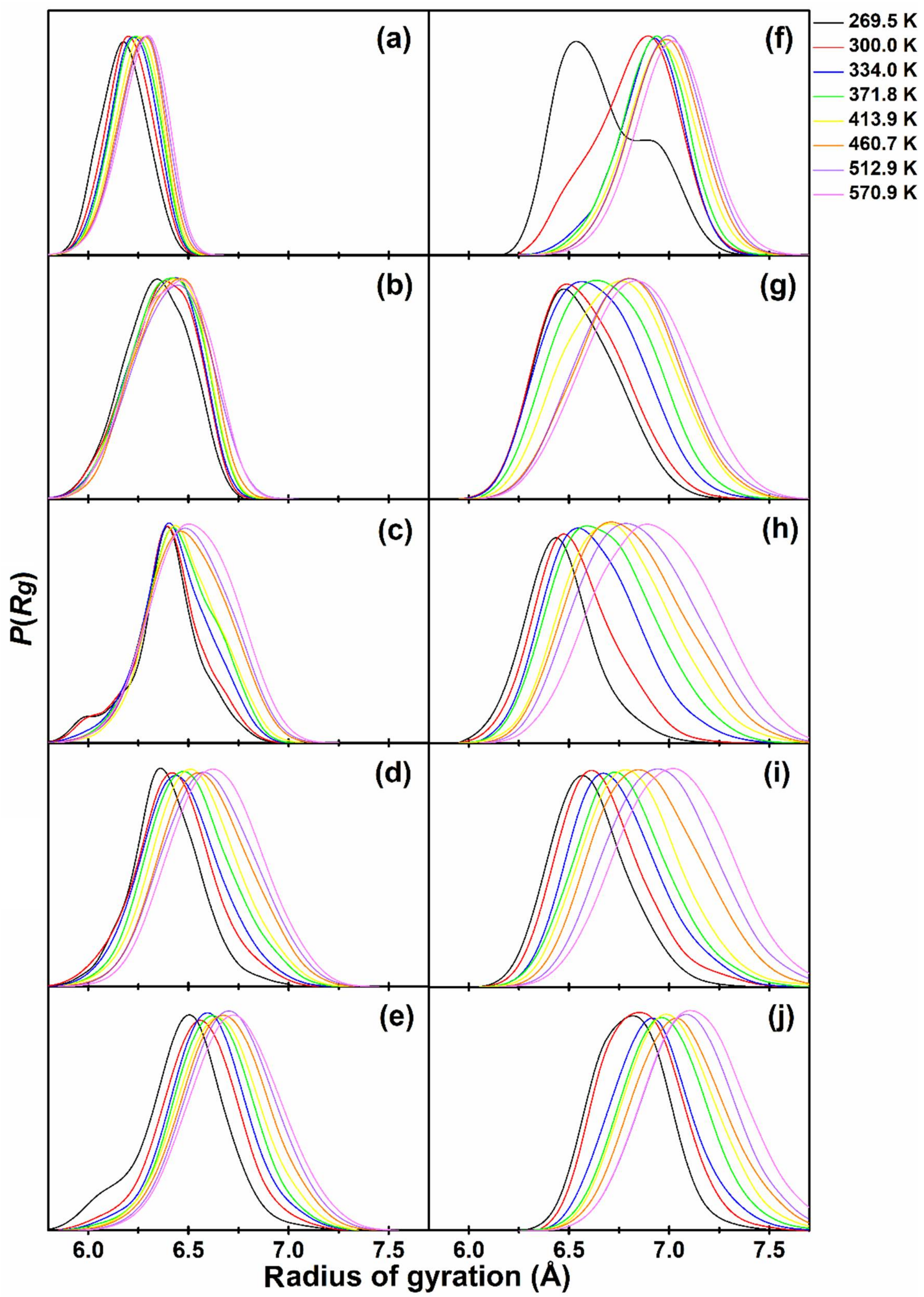

Figure S13. The radius of gyration in various temperatures for (**a**) βCD, (**b**) Mon6-HPβCD, (**c**) Di6-HPβCD, (**d**) Tri6-HPβCD, (**e**) Tet6-HPβCD, (**f**) Hep6-HPBCD, (**g**) Mon2Tet6-HPBCD, (**h**) Di2Tet6-HPBCD, (**i**) Tri2Tet6-HPBCD, and (**j**) Tet2Tet6-HPBCD.

Table S1. The probability of different numbers of flip glucose subunits in βCD and all HPβCDs using flip angle parameter, $\theta_{(i)}[C6_{(i)}–C2_{(i+1)}–C6_{(i+1)}]$ in the various temperatures (criteria: more value than 90 degree) compared with classical MD simulation in the parenthesis.

The Percentage of Flip Angle (%)												
**Temperature**	**269.5 K**			**300.0 K**			**334.0 K**			**371.8 K**		
**Model**	**No flip**	**One flip**	**Two flips**	**No flip**	**One flip**	**Two flips**	**No flip**	**One flip**	**Two flips**	**No flip**	**One flip**	**Two flips**
**βCD**	52	38	10	58 (28)	35 (53)	7 (19)	61	33	6	59	34	7
**Single-sided HP substitution**												
**Mon6-HPβCD**	65	32	3	69	28	3	68	29	3	67	29	4
**Di6-HPβCD**	75	23	2	74	23	3	76	22	2	76	22	2
**Tri6-HPβCD**	70	29	1	75	24	1	78	21	1	77	21	2
**Tet6-HPβCD**	66	33	1	78	21	1	81	18	1	81	18	1
**Hep6-HPβCD**	43	54	3	73 (84)	25 (15)	2 (1)	81	18	1	79	19	2
**Double-sided HP substitution**												
**Mon2Tet6-HPβCD**	77	21	2	77	22	1	77	22	1	74	24	2
**Di2Tet6-HPβCD**	67	32	1	75	24	1	78	20	2	78	20	2
**Tri2Tet6-HPβCD**	67	31	2	70	28	2	71	26	3	68	28	2
**Tet2Tet6-HPβCD**	74	23	3	74	24	2	74	24	2	73	24	3
**Temperature**	**413.9 K**			**460.7 K**			**512.9 K**			**570.9 K**		
**Model**	**No flip**	**One flip**	**Two flips**	**No flip**	**One flip**	**Two flips**	**No flip**	**One flip**	**Two flips**	**No flip**	**One flip**	**Two flips**
**βCD**	63	30	7	63	30	7	62	31	7	61	32	7
**Single-sided HP substitution**												
**Mon6-HPβCD**	65	30	5	65	30	5	64	30	6	61	32	7
**Di6-HPβCD**	73	24	3	71	26	3	66	29	5	65	29	6
**Tri6-HPβCD**	76	21	3	73	24	3	68	28	4	64	30	6
**Tet6-HPβCD**	78	20	2	76	21	3	73	24	3	67	28	5
**Hep6-HPβCD**	77	21	2	73	23	4	69	26	5	64	30	6
**Double-sided HP substitution**												
**Mon2Tet6-HPβCD**	74	24	2	73	24	3	70	26	4	65	29	6
**Di2Tet6-HPβCD**	76	22	2	72	25	3	69	27	4	66	29	5
**Tri2Tet6-HPβCD**	68	29	3	68	28	4	68	28	4	66	29	5
**Tet2Tet6-HPβCD**	72	25	3	70	27	3	67	29	4	65	30	5

Table S2. The probability of 25,000 snapshots with different numbers of HP occupied in CD cavity (criteria: $d_{4(i)}$[$C_{g(\beta CD)}$–$C_{g(HP(i))}$] < 3 Å) for all HPβCDs in the various temperatures.

The Probability of Different Numbers of $d_{4(i)}$]$C_{g)\beta CD)}$–$C_{g)HP(i))}$] < 3 Å (%)									
**Model**	n(**HP**$_{inserted}$)	**Temperature (K)**							
		**269.5**	**300.0**	**334.0**	**371.8**	**413.9**	**460.7**	**512.9**	**570.9**
**Single-sided HP substitution**									
**Mon6-HPβCD**	**0**	96.45	97.59	97.88	97.25	97.81	98.80	98.63	98.33
	**1**	3.55	2.41	2.12	2.75	2.19	1.20	1.37	1.67
**Di6-HPβCD**	**0**	84.44	86.80	91.21	93.00	94.30	94.69	95.39	96.01
	**1**	15.56	13.20	8.79	7.00	5.70	5.31	4.61	3.99
**Tri6-HPβCD**	**0**	51.92	62.69	71.25	77.77	84.25	88.56	90.54	94.16
	**1**	48.06	37.25	28.72	22.22	15.75	11.44	9.44	5.84
	**2**	0.02	0.06	0.03	0.01	0.00	0.00	0.02	0.00
**Tet6-HPβCD**	**0**	46.44	61.66	68.80	74.52	77.54	83.42	87.17	89.24
	**1**	53.48	38.30	31.13	25.46	22.42	16.58	12.82	10.76
	**2**	0.08	0.04	0.07	0.02	0.04	0.00	0.01	0.00
**Hep6-HPβCD**	**0**	15.14	42.41	61.04	65.90	74.98	79.94	83.04	85.76
	**1**	84.59	57.45	38.88	34.06	24.99	20.02	16.92	14.21
	**2**	0.27	0.14	0.08	0.04	0.03	0.04	0.04	0.03
**Double-sided HP substitution**									
**Mon2Tet6-HPβCD**	**0**	42.64	44.28	51.70	58.09	67.69	72.06	76.31	79.21
	**1**	57.04	54.53	47.42	41.37	31.98	27.73	23.50	20.66
	**2**	1.32	1.19	0.88	0.54	0.33	0.21	0.19	0.13
**Di2Tet6-HPβCD**	**0**	23.51	33.40	45.25	51.01	56.64	60.77	65.14	71.96
	**1**	75.37	65.36	53.95	48.22	42.68	38.77	34.46	27.75
	**2**	1.12	1.24	0.80	0.77	0.68	0.46	0.40	0.29
**Tri2Tet6-HPβCD**	**0**	21.11	26.46	34.52	39.18	43.60	53.79	60.66	67.70
	**1**	77.73	72.24	64.44	59.93	55.42	45.59	38.83	31.99
	**2**	1.16	1.30	1.04	0.89	0.98	0.62	0.51	0.31
**Tet2Tet6-HPβCD**	**0**	35.34	38.58	40.27	47.16	48.88	51.23	53.28	60.79
	**1**	63.94	60.66	58.84	52.22	50.36	48.10	46.10	38.84
	**2**	0.72	0.76	0.89	0.62	0.76	0.67	0.62	0.37
